# Is There Need for Pancreatic Enzyme Replacement Therapy in Patients with Exocrine Pancreatic Insufficiency When Using High-Caloric Liquid Diets? Orientating Studies on Praecaecal Digestibility in Pigs with Experimentally Induced Pancreatic Exocrine Insufficiency and Ileocaecal Fistula

**DOI:** 10.3390/biom15101392

**Published:** 2025-09-30

**Authors:** Anne Katrin Mößeler, Annette Liesegang, Paul Torgerson, Josef Kamphues

**Affiliations:** 1 Institute for Animal Nutrition, University of Veterinary Medicine Hannover, Foundation, D-30173 Hanover, Germany; 2 Institute of Animal Nutrition and Dietetics, Vetsuisse Faculty, University of Zurich, CH-8057 Zürich, Switzerland; aliese@nutrivet.uzh.ch; 3 Section of Epidemiology, Vetsuisse Faculty, University of Zurich, CH-8057 Zürich, Switzerland

**Keywords:** malnutrition, pancreatic exocrine insufficiency, pancreatic enzyme replacement therapy, protein, fat, high-calorific liquid supplements, animal model

## Abstract

In patients with pancreatic exocrine insufficiency (PEI), focus is primarily placed on fat digestion. Using high-caloric drinks (HCD) is often recommended to avoid malnutrition, but knowledge is limited whether pancreatic enzyme replacement therapy (PERT) is needed. In this study the animal model of pancreatic duct-ligated (PL) and ileocaecal-fistulated minipig was used to determine the praecaecal disappearance rates (pcDR) of the fat and protein of four HCD in controls and PL-pigs with or without PERT. In controls pcDR were high (95.5–96.6% for fat; 70.2–78.6% for protein) while in PL-pigs receiving no PERT the pcDR were significantly lower (fat DR: 47.4–54.3%; protein 22.4–33.5%) despite a high fat pcDR value (84.0%) of one diet. PERT resulted in a normalisation of pcDR of fat and protein with values not differing from controls. This study demonstrates the massive impact of PEI on pcDR, even in HCD typically considered highly digestible. Using PERT is highly recommended in PEI patients using HCD to avoid maldigestion and associated digestive tract symptoms. Optimisation of formulations and galenic preparations of the HCD seems to be necessary as well, as the high fat pcDR of one drink showed that even without PERT high values can be reached.

## 1. Introduction

Pancreatic exocrine insufficiency (PEI) is a maldigestion syndrome relevant to both human [1] and veterinary medicine. According to the European guidelines for the diagnosis and treatment of PEI [2], PEI is defined as follows: “A reduction in the exocrine pancreatic secretion and/or intraluminal activity of pancreatic enzymes below the level that allows normal digestion of nutrients. PEI is associated with the malabsorption of nutrients and may result in intestinal symptoms and/or nutritional deficiencies.” The aetiology of PEI is diverse. While chronic pancreatitis (CP) and cystic fibrosis (CF) are common causes, acute necrotising pancreatitis, pancreatic cancer, anatomical changes following surgery, and other digestive tract disorders have also been reported as underlying factors. Therefore, pancreatic exocrine insufficiency must be considered a maldigestion syndrome rather than an isolated organ defect [2]. Typical symptoms of PEI are bloating, steatorrhea, and weight loss as well as deficiency in fat-soluble vitamins [1,2,3,4], affecting health status but also the quality of life of the patients.

The standard treatment and cornerstone in the treatment of PEI [1,2,3,4]—regardless of aetiology—is pancreatic enzyme replacement therapy (PERT), which is performed most frequently using porcine-derived enzymes, although non-porcine alternatives are now available in some countries. Small-sized, enteric-coated pellets are the preferred pancreatin preparations for PEI. The therapeutic goal is the resolution of nutritional deficiencies and the relief of symptoms and signs associated with PEI [2,4]. Enzyme dosage, with or without the addition of a proton pump inhibitor (PPI), should be tailored to the individual [5]. PERT is usually titrated, with the aim of reducing or eliminating steatorrhea [3], but it is worth mentioning that only 71% of international experts prescribes PERT in the case of steatorrhea [6]. PEI ought to be addressed in alignment with recognised clinical guidelines [2,7]. In one study [3], the median PERT dosage used was 96,000 units of lipase with each meal to treat PEI, while a recently published recommendation [4] aims for at least 40,000 USP units lipase per meal in adults and 20,000 USP units lipase per snack.

However, Rijk et al. showed in a recent study [6] based on an international expert survey that despite various published guidelines there is still a considerable lack of consensus and variation in the management of EPI patients with CP. In many malnourished patients with PEI, the use of high-calorific supplemental nutrition is standard practice to ensure adequate nutrient intake. Most human digestibility studies rely on stool samples, which do not distinguish between praecaecal and post-ileal digestive processes. Because post-ileal fat digestion is minimal, stool fat analysis is reliable for assessing fat digestion. However, for protein and starch, post-ileal fermentation can lead to a considerable overestimation of praecaecal digestibility (predominantly enzymatic digestion) and nutritive value [7,8,9,10,11,12,13].

Post-ileal crude protein (respectively, N) absorption is of limited benefit—it primarily occurs in the form of ammonia absorption, which is more of a metabolic burden than a nutritional advantage. Therefore, knowledge of praecaecal protein digestion is essential [8,11]. Animal models that allow sampling of digesta at the terminal ileum, in addition to faeces, make it possible to differentiate between enzymatic digestion (praecaecal) and microbial fermentation in the hindgut [8]. In animal nutrition, praecaecal protein digestibility is a global standard for evaluating the nutritional value of protein sources.

Although clinical recommendations for dietary management and PERT in PEI mainly focus on fat digestion and lipase supplementation [2,3], experimental studies in animal models have shown that protein and starch digestibility rates are also impaired [8,10,13,14]. Low praecaecal protein digestibility may contribute to sarcopenia, muscle mass loss, and reduced muscle strength. A recent study [15] demonstrated the importance of adequate PERT in patients with advanced pancreatic cancer to prevent muscle loss. Notably, low-dose PERT (75,000 IU lipase/day) was independently associated with higher odds of muscle loss compared with high-dose therapy—regardless of tumour response, disease stage, or chemotherapy regimen. Patients with PEI may be undertreated [4,5,6,16]. This highlights the need for sufficient enzyme replacement [4,17,18], with attention not only given to lipase but also to protease supplementation. In the European guidelines for the diagnosis and treatment of pancreatic exocrine insufficiency [2], the occurrence of protein deficiency in case of PEI is also named and high protein food is recommended for EPI patients [4].

Furthermore, a study using the animal model of PEI has demonstrated increased endogenous protein losses [19]. Marked changes in functional proteins may occur in PEI patients. Retinol-binding protein and prealbumin may help to monitor PERT effectiveness in PEI patients [20]. PEI affects these parameters, and maintaining normal levels and functionality of these proteins depends on effective PERT. Currently, published clinical studies evaluating the efficacy and safety of protease enzyme products administered with high-calorie beverages for the prevention of malnutrition are limited.

## 2. Materials and Methods

### 2.1. Aim of the Study

This experimental investigation sought to evaluate whether praecaecal digestibility is affected in cases of experimentally induced pancreatic exocrine insufficiency (PEI) when high-calorie liquid supplements are administered. Various diets were used to assess potential differences regarding praecaecal digestibility between products. The actual guidelines for the diagnosis and treatment of pancreatic exocrine insufficiency [2] mention in statement 3.9.2 that “if required, PERT can be added to enteral nutrition; however, its efficacy has not yet been proven”. On the other hand, the ESPEN-ESPGHAN-ECFS guidelines on nutrition care for cystic fibrosis [7] state that while a high-calorie, high-fat diet with pancreatic enzyme replacement therapy (PERT) and fat-soluble vitamin supplementation was the standard of nutritional care for CF for decades, no precise information was included regarding high-calorific supplements (drinks). This study also aimed to assess whether PERT is beneficial or even crucial with commercial high-calorific liquid formulas. The focus was placed not only on fat digestion but also on protein digestion, given that protein maldigestion appears to be an underestimated problem in PEI patients [21]. In this orientating study, a high dose PERT was tested as this study aimed to test the principle in general. Therefore, this study did not aim to establish recommendations regarding PERT dosage for PEI patients using HCD.

### 2.2. Materials and Methods

The studies were conducted using an animal model involving pancreatic duct ligation and ileocaecal fistulation in minipigs. This is a well-established model for investigating the effects of pancreatic exocrine insufficiency (PEI) and pancreatic enzyme replacement therapy (PERT) [10,14,22,23,24,25,26]. The ileocaecal fistula enables the collection of ileal chyme, allowing the quantification of the praecaecal digestibility of nutrients, which is particularly important for assessing starch and protein digestion [10,13].

### 2.3. Animals

Every effort was undertaken to minimise animal stress and to limit the number of minipigs included in this study. The trial was conducted in accordance with the German animal welfare regulations and with the European Council Directive of 24 November 1986 (86/609/EEC) and was approved by the Ethics Committee of Lower Saxony for the Care and Use of Laboratory Animals (LAVES: Niedersächsisches Landesamt für Verbraucherschutz und Lebensmittelsicherheit; reference: 33.9-42502-04-15/1910). This study used the minimum number of animals required under the 3-R principle and German animal welfare rules.

All animals underwent acclimatisation to handling and sample collection procedures and exhibited no observable indicators of stress or discomfort. Overall, nine adult female Ellegaard^®^ (Dalmose, Denmark) minipigs, fitted with an ileocaecal canula (method described by Tabeling [27]), were used in this study. In five of these animals, PEI was experimentally induced by ligation of the ductus pancreaticus accessorius (PL); the other four with normal exocrine pancreatic function were used as controls (CON). Faecal chymotrypsin concentration was measured in all animals (test kit purchased from Immundiagnostik AG, Wiesenstrasse 4, 64625 Bensheim, Germany, catalogue number K6990) and only minipigs with a chymotrypsin activity < 0.900 U/g faeces were defined and used as PEI-pigs. In the present study the body weight of the minipigs enrolled was 30.7 ± 4.74 kg (Con) and 27.1 ± 1.21 kg (PL). The animals underwent surgery at least 8 months before the experiments of this study started.

### 2.4. Liquid Test Diets—Commercial High-Calorific Drinks (HCDs) for Human Consumption

The liquids diets tested in this test were HCDs designed for enteral nutrition of patients with maldigestion and contained at least 1.5 kcal/mL. Each liquid supplement was fed to every animal in a Latin square design, and the animals underwent each trial twice—one time without PERT and one period with a porcine pancreatic enzyme product at a dosage of 300,000 I.U. lipase (accounting for 20,600 I.U. protease per meal). Additionally, all liquid diets were tested in four healthy minipigs with normal exocrine pancreatic function, which were used as controls. The test was conducted as a screening procedure based on the methodology established by Becker [28] and Classen [29], and accordingly, the term “disappearance rate” (DR) was employed in place of “digestibility rate” as referenced by Mößeler et al. 2007 [8]. Phases without PERT for the PL-pigs were reduced to a minimum (this was generally performed 24 h before the screening test to avoid any influence on test results and the screening test itself if PL animals underwent the trial without PERT).

For this study four commonly used liquid nutritional supplements (high-calorific drinks (HCDs)) from different suppliers were selected from a local pharmacy in Germany according to the local selling figures in the year 2016. Of these four HCDs, two (A and B) were rich in fat (9.3 g/100 mL) compared to values of 4.9 and 5.8 g/100 mL in the other diets. Protein content was moderate (5.6 and 6.25 g/100 mL) in two of these HCDs with higher values in the other ones used (9.6 and 10.2 g/100 mL). Among these HCDs, two had an energy density of 1.5 kcal/mL, while the other two (A and B) had an energy density of 2.4 kcal/mL. The nutritional key points of these HCDs are given in Table 1.

Each liquid diet was given at an amount of 800–850 mL per meal and nutrient intake was calculated from the analysed values of the diets (including 30 g Methocel™ (Hydroxypropyl-Methylcellulose, Sigma Aldrich Chemie, Taufkirchen, Germany). For two HCDs (A and B), the protein intake was around 81 resp. 85 g while the other HCD resulted in a total intake of around 44 and 50 g (see Table 2).

### 2.5. Test Design

The tests were performed as a screening test established by Becker [28] and Classen [29] to test the efficacy of PERT in the animal model of the ileocaecal-fistulated and pancreatic duct-ligated minipig. The different test diet drinks (including 0.368 g Cr_2_O_3_ (Sigma Aldrich Chemie, 98% ≤ 50 μm) as a marker to calculate disappearance rates) were fed only once (no adaptation period) and chyme collection was performed over 8 h after the first occurrence of the green colour of the chyme indicated the arrival of the test diet at the fistula at the terminal ileum [27]. To ensure a parallel flow of nutrients and marker, 30 g methylcellulose (Methocel™, Sigma Aldrich Chemie, Taufkirchen, Germany) was added to each meal. When animals were included in a trial, the maintenance therapy (regular administration of porcine multienzyme product) for PL-pigs was discontinued the last day prior to the test. At the evening meal before the test, the pigs were fed only 400 mL of a liquid diet (ProvideXtra DRINK™ by Fresenius Kabi Deutschland GmbH, Bad Homburg, Germany) to optimise gastric emptying and to avoid a carryover of nutrients of the diet from the previous evening into the test [28,29]. A period of at least 48 h was performed between two screening tests to ensure a washout of Cr_2_O_3_ out of the praecaecal gastrointestinal tract. The different liquid test diets were tested in a randomised order with every pig receiving all diets. The control pigs received each test diet once while the PL-pigs underwent the test of each liquid diet twice—one time without PERT and one time with PERT. A coated commercial pancreatic enzyme product of porcine origin was administered at a dosage of 300,000 I.U. lipase (containing 20,600 I.U. protease) per meal. The enzymes were added to the liquid diet. Test diets were taken up rapidly and completely by all pigs.

### 2.6. Sampling

The ileal chyme was sampled by opening the re-entrant fistula, closing the caecal fistula with a metallic cover containing a membrane. Positioning of flexible bins at the ileal fistula allowed collection of the ileal chyme.

The bins were regularly serviced, with frequent emptying and replacement as necessary. Substitution of water and electrolytes at an amount of 1 d/g collected chyme) was carried out via the membrane of the caecal fistula every two hours according to the method described by Tabeling [29].

### 2.7. Sample Preparation and Analyses

Sampled ileal chyme was weighed immediately after collection using an analytical scale and then frozen at −20 °C. Afterward, the samples were freeze-dried (freeze dryer: alpha 1–4 LSC, Martin Christ, Gefriertrocknungsanlagen GmbH, Osterode am Harz, Germany), and analysed regarding dry matter, crude fat, and crude protein according to reference [30]; crude fat content was determined by acid hydrolysis and petrol ether extraction using a filter bag technique in an extractor (ANKOM XT15; ANKOM Technology, Macedon, NY, USA) for protein combustion after Dumas. Cr_2_O_3_ was determined according to the method of Petry and Rapp [31]. The apparent praecaecal disappearance rate (pcD) was calculated according to Becker [27]. For fat as well as protein, the values given represent the apparent digestibility, i.e., the disappearance rate.

### 2.8. Statistical Analyses

The *t* test was used to compare the praecaecal disappearance rate of fat and protein between controls, PL and PL + PERT groups. Alternatively, where there was evidence of significant departure of the data from a normal distribution, comparisons were made using a Wilcoxen test. All analysis was undertaken in R (R Core team, 2024; R version 4.3.3, R Foundation for Statistical Computing, Vienna, Austria, https://www.R-project.org).

## 3. Results

### 3.1. Effect of PEI and PERT on pcDR of Fat

**Figure 1:**
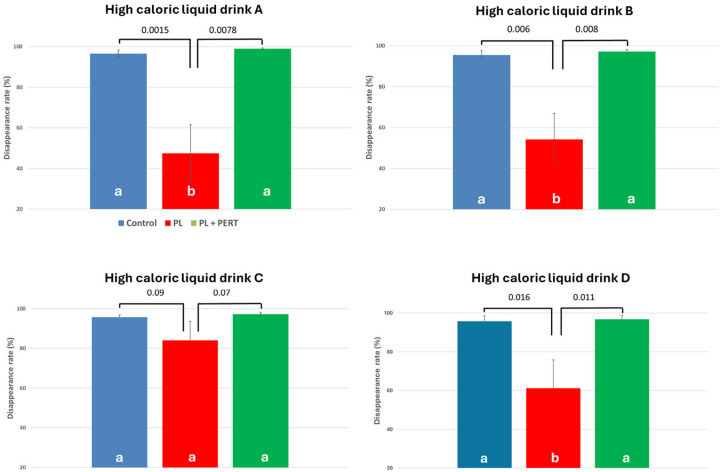
(**A**–**D**) Praecaecal disappearance rate of fat in CON (blue bar), PL (red bar) and PL + PERT (green bar) group when different high-calorific liquid diets were fed. Different letters mark significant differences between the treatment groups.

The fat pcDR was very high (varying between 95.5% ± 2.20 (HCD B) and 96.6% ± 1.75 (HCD A)) in CON (see Figure 1A,B). PL-pigs showed a significantly lower fat pcDR with values varying between 47.4% ± 14.2 (HCD A) and 54.3% ± 12.6 (HCD B) for three of the HCD tested. There was one exception: the fat pcDR estimated for HCD C was quite high (84.0% ± 9.60) in PL-pigs and the value did not differ significantly from that observed in CON. PERT resulted in a marked increase in fat pcDR with values not significantly differing from CON (being even numerically higher), resulting in values between 96.6 ± 1.17 (HCD D) and 99.0 ± 0.365 (HCD A).

### 3.2. Effect of PEI and PERT on Apparent pcDR of Crude Protein

**Figure 2:**
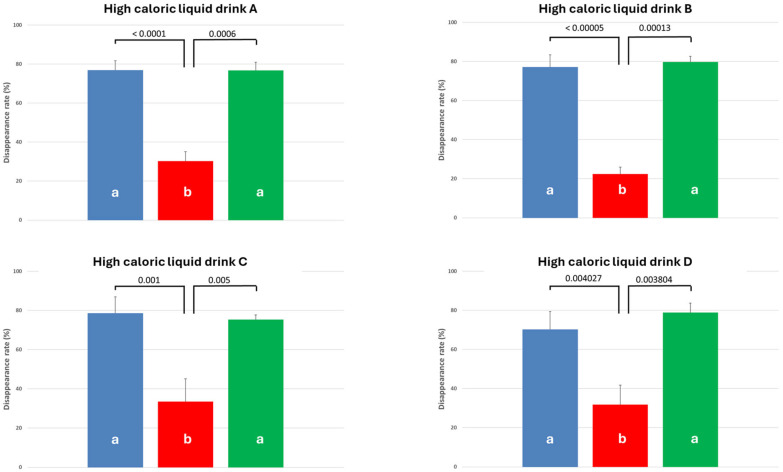
(**A**–**D**) Apparent praecaecal disappearance rate of protein in controls (blue bar), PL (red bar), and PL + PERT (green bar) group when fed different high-calorific liquid diets. Different letters mark significant differences between the treatment groups.

The apparent pcDR of crude protein was rather high (varying between 70.2% ± 9.19 (HCD D) and 78.6% ± 8.43 (HCD C) in CON (see Figure 2)). For all HCDs, the PEI-pigs showed a significantly lower protein pcDR with values varying between 22.4% ± 3.35 (HCD B) and 33.5% ± 11.6 (HCD C). PERT significantly increased protein pcDR in PL-pigs, with results comparable to CON and even numerically higher for HCD B and D, ranging from 75.3% ± 2.13 (HCD C) to 79.8% ± 2.92 (HCD B).

### 3.3. Effect of Liquid Drink on DR of Nutrients

Crude fat pcDR (%): No significant differences were found for either the controls or the PERT group between the tested HCDs regarding the pcDR of crude fat. (see Table 3) In the PL group, values varied between 47 and 54%, while for one HCD (C), the estimated crude fat pcDR was significantly higher, reaching 84%.

Crude protein DR: No significant differences for apparent pcDR of protein were observed between the four HCD (comparing the values within one treatment group (CON, PL, PL + PERT)). The values did not differ between different diets; for all diets, the values observed in the control and PERT group varied between 70 and 80%, while the protein of all diets was digested to a lower rate of 22 to 32% in the PL group (see Table 4).

## 4. Discussion

### 4.1. Critical Discussion of the Methods Used

Even though this study was performed in a rather low number of animals, since fistulated animals were used, the results show clear the effects of PEI and PERT on pcDR of fat and protein. The fact that each diet was tested in identical animals and each individual with PEI underwent the tests twice (with or without PERT) minimised individual effects. As expected, the variation was lower in the controls and in the PL animals receiving PERT.

The fixed enzyme dosage used was rather high with 300,000 IU lipase per test meal; as the amount of fat differed between 42 g up to 77 g, the ratio of lipase per g fat varied between 7100 and 3900 IU lipase per g fat. The decision to use a fix dose of enzyme may be questionable—but as a commercial multienzyme product was used, the adaptation of the enzyme dosage according to the fat content of the meal would not result in a comparable protease dosage per g protein either. As this study was an orientating study with a focus on the question of whether enzymes are needed for HCD in patients with PEI and whether the normalisation of digestion is possible in the case of high enzyme dosage, the fixed dosage was chosen.

The amount of HCD per test meal was rather high (800 mL), exceeding typical meal sizes in human patients, resulting in a high nutrient intake, and causing a quite challenging situation regarding digestive processes, as the digestive capacity may have been exhausted. The high volume of the test drink was needed to ensure collection of sufficient amounts of ileal digesta to perform the chemical analyses. The fact that in controls the pcDR was almost complete (above 95% for crude fat for all diets and above 75% for protein in all diets but one) shows that a healthy individual can cope with that amounts of nutrients per meal. As PERT resulted in a normalisation of digestive processes in PL-pigs, with values not differing from controls, it can be concluded that neither enzyme dosage nor absorptive capacity was a limiting factor in this study.

### 4.2. Clinical Relevance of This Study

An impaired digestion of nutrients is a typical symptom of PEI and is well known for complex meals, but knowledge about HCDs designed to prevent malnutrition is limited, with only few studies available [32]. As expected, the tested HCDs were highly digestible in CON animals with values varying between 95 and 97% for fat and around 70 to 79% for crude protein. On the other hand, the pcDR of fat was, in general, much lower in PL-pigs without PERT but also showed much more variation between the different HCD used. For crude protein the pcDR values were distinctly lower in PL-pigs compared to controls. High-dosed PERT was able to cause a significant improvement and normalisation of pcDR for fat as well as protein, reaching the values of the controls.

As many studies in PEI patients are focussed on fat [32], protein maldigestion resulting in protein deficiency may be an underestimated problem. Protein deficiency may cause muscle loss and impair many functions, as many important molecules in metabolism are proteins [15]. A study recently performed in mice [33] showed that deficiency of dietary protein and amino acids can inhibit the mRNA translation of pancreatic digestive enzymes and therefore PEI and malnutrition can be induced. From the findings of that study and the findings from our own investigations (massively impaired protein digestibility in PEI), it can be assumed that an untreated PEI may occur due to protein deficiency.

### 4.3. Consequences for Practical Recommendations for Nutritional Support for PEI Patients: Does the Labelled Nutrient Content Reflect the Real Nutrient Uptake?

Furthermore, the present results underline the difficulties of nutritional recommendations for PEI patients, as the nutrient intake may be quite different from the nutrient absorption [9]. To clarify this aspect, a calculation of the amount of digested nutrients and comparison with the intake on 100 mL basis was performed. By considering the pcDR and the nutrient content of the HCD, the nutrient uptake per 100 mL can be calculated (see Table 5). While for CON and PL + PERT the correlation between nutrient intake and digested nutrients was quite good and 95 to 99% of crude fat that was ingested was digested, the ranking of the digested amount of crude fat did not correlate nicely to the crude fat intake in PL. For the two HCDs (A, B) with higher fat content (9.3 g/100 mL), PL-pigs without PERT showed pcDR for fat varying from 47% (HCD A) to 54% (HCD B) while the HCD with lower fat content (HCD C, D) showed comparable (53%; HCD D) or higher values (84%; HCD D). When the total amount of praecaecally-digested crude fat was calculated, the two HCDs (C and D) with 5.8 resp. 4.9 g crude fat per 100 mL had marked differences regarding digested fat (4.87 g/100 mL for HCD C and 2.59 g/100 mL for HCD D).

It needs to be considered that in PL group the HCD B (with high crude fat content of 9.35 g per 100 mL) and HCD C (5.8 g crude fat content per 100 mL) resulted in a comparable amount of digested fat (5.1 g per 100 mL for HCD B and 4.9 g per 100 mL for HCD C). On the other hand, the high DR of the crude fat in HCD C resulted in a much better utilisation of fat from this diet. If only declared values were considered, HCD C’s benefit would be underestimated. Taking this into account, it is obvious that conclusions cannot be drawn directly from the labelled nutrient content to the nutrient intake. The fact that HCD C showed a much better DR leads to the question of whether this can be assumed by the declared composition. As in the other HCDs, oils of plant origin were the basis of this HCD, showing markedly higher pcDR and therefore higher benefit for the patient. As observed in an earlier study using this animal model the fat source, i.e., the fatty acid composition, markedly affects digestibility in PL individuals [11,34]. The melting point of fat had a distinct effect on digestibility of PEI (the lower the melting point, the higher the digestibility rates of the fat in PL-pigs). It can be assumed that beside chemical composition, the preparation or technical aspects are relevant (e.g., emulsification). Considering the labelled ingredients of the tested HCD, the information is limited, as all HCDs contained milk protein and oils of vegetable origin (see Table 6). As all HCDs contained rapeseed and sunflower oil as fat sources (with HCD B and D containing also corn oil), it can be speculated that emulsification is of great interest and may explain the differences in pcDR. Therefore, the fatty acid composition and/or grade of emulsification is assumed to be important for the digestibility of fat and should be in focus when these high-calorific diets are formulated and optimised. For future studies, it seems therefore reasonable to do more precise analyses and characterise the fat and emulsification grade of the HCD.

When looking at the situation regarding the crude protein intake, the differences were smaller (see Table 7). Regarding protein, two of the four HCDs used in this study were higher in protein (9.6 (HCD A) and 10.2 (HCD B) g per 100 mL) than the others (5.6 to 6.3 g). Nonetheless, the HCD with the highest protein content (HCD C, 10.2 g/100 mL) resulted only in a slightly higher (2.29 g vs. 1.88 g/100 mL) amount of digested protein compared to the one (HCD D) with a distinctly lower protein content (5.6 g protein/100 mL). Additionally, even though the crude protein intake with HCD B (10.2 g/100 mL) was markedly higher compared to HCD D (3.55 g per 100 mL drink), the amount of digested crude protein was only 0.28 g/100 mL higher in PL (see Table 7). Therefore, for protein—same as for fat—the labelled nutrient content does not allow to calculate the amount of digested nutrients in the case of untreated PEI: When PERT was used, the labelled fat and protein content and the amount of digested nutrients matched quite well.

To sum up, PEI without PERT resulted in a distinct reduced pcDR of fat and protein. The finding of reduced fat digestion is well known and typical for PEI. Furthermore, the use of the animal model of pancreatic duct-ligated and ileocaecal-fistulated pig allows the study of protein digestion in the case of PEI [8,10,13,14,19], where praecaecal digestion was also impaired markedly.

This study clearly underlines the need to use PERT when HCDs are used to prevent malnutrition and to optimise nutritional status although the present data does not allow to give any recommendations regarding enzyme dosage needed. The use of PERT is of special interest as low praecaecal digestibility results in post-ileal fermentation, causing bloating, flatulence, and pain as well as steatorrhea and osmotic diarrhoea. PERT is quite easy to implement when patients are ingesting the HCD orally—but much more challenging when patients receive liquid diets during the night via a percutaneous endoscopic gastrostomy (PEG) tube. The recommendation of the ESPEN-ESPGHAN-ECFS guideline on nutrition care for cystic fibrosis [7] states that “Pancreatic enzymes can be administered orally at the start of enteral nutrition (EN) and during the night if the patient is awake. Enzymes in non-enteric or powder form can be mixed in with the feed if oral intake of enzymes is not possible.” This orientating study showed clearly that enzymes are needed for HCD in patients with PEI to normalise digestive processes and should be used whenever possible. These results also show clearly that the benefit of using HCD for the patients depends not only on the labelled nutrient content, but also on the digestibility. As undigested nutrients may cause digestive disorders and impair the wellbeing of the patient, this needs to be considered. An interesting finding was the marked difference between the different HCD tested—while most diets showed comparable results, one HCD showed a much higher pcDR for fat than the others. This result indicates that the HCDs differ regarding the digestibility and that tests for evaluation and optimisation are needed. The animal model used in this study is very valuable to evaluate the effects of PERT and to test different diets; nonetheless, the need for fistulated animals and the quite high level of care limits studies. However, another model that was used to evaluate the protein digestion was described previously [35] and might be an additional interesting method to assess protein digestibility.

## 5. Conclusions

This experimental study shows the marked effects of PEI on pcDR of fat as well as protein. High-dosed PERT was able to cause a complete normalisation of digestion in individuals with PEI while the HCDs were only digestible to a low extent when no PERT was given (with one exceptional high pcDR of fat for one HCD). The fact that one HCD showed a quite high pcDR of fat when given without PERT raises the question whether HCD can be optimised regarding components and galenic preparation to achieve a high pcDR of fat even without PERT. The pcDR for protein were rather low in PL-pigs receiving no PERT, underlining the need to take protease supplementation into account in the case of PEI.

In conclusion, for the praxis we recommend that different HCDs should be used and the clinical outcome (weight gain, symptoms of the digestive tract due to undigested nutrients entering the hindgut causing bloating or steatorrhea) should be observed carefully. But it must be kept in mind that when high-dosed PERT was used, the values of digestibility were high for all HCDs used in this study and did not differ from those of the controls. It can be assumed that even lower-dosed PERT improves digestibility of nutrients of these HCDs. Therefore, it seems reasonable and necessary to recommend multienzyme PERT whenever HCDs are used in patients with PEI. Although the translation of results from an animal study into clinical practice must always be carried out carefully, this orientating study in the animal model may help to deepen the knowledge and understanding of the need for PERT in PEI patients. This is of special interest as the percentage of international experts that recommend PERT [4] is surprisingly low.

## Figures and Tables

**Table 1:** Nutrient content (g) as well as energy density (per 100 mL) of the commercial high-calorific liquid products designed for enteral nutrition to prevent malnutrition used in this study.

HCD	Fat (Total)	Fat Saturated	Protein	kJ/kcal
A	9.3	0.9	9.6	1010/240
B	9.35	0.83	10.2	1008/240
C	5.8	0.4	5.6	630/150
D	4.9	0.5	6.25	630/150

**Table 2:** Amount of nutrients per test meal using the commercial high-calorific liquid products designed for enteral nutrition to prevent malnutrition used in this study.

HCD	Dry Matter Intake (g)	Fat Intake (g)	Protein Intake (g)
A	414	75	81
B	422	77	85
C	260	47	44
D	273	42	50

**Table 3:** Apparent praecaecal disappearance rate (%) of fat in controls, pancreatic duct-ligated (PL) pigs, and PL-pigs receiving high-dosed pancreatic enzyme replacement therapy.

HCD	CON	PL	PL + PERT
A	96.6 ± 1.75 a	47.4 ± 14.2 a	99.0 ± 0.365 a
B	95.5 ± 2.20 a	54.3 ± 12.6 a	97.2 ± 1.05 a
C	95.7 ± 1.20 a	84.0 ± 9.60 b	97.3 ± 0.820 a
D	96.4 ± 1.47 a	52.8 ± 6.01 a	96.6 ± 1.17 a

Different letters mark significant differences between the tested liquid diets within one group of animals (in one column).

**Table 4:** Apparent praecaecal disappearance rate (%) of protein in controls, pancreatic duct-ligated (PL) pigs, and PL-pigs receiving high-dosed pancreatic enzyme replacement therapy.

HCD	CON	PL	PL + PERT
A	76.9 ± 4.88	30.2 ± 4.89	76.7 ± 4.26
B	77.1 ± 6.27	22.4 ± 3.53	79.8 ± 2.92
C	78.6 ± 8.43	33.5 ± 11.6	75.3 ± 2.31
D	70.2 ± 9.19	31.9 ± 9.93	78.8 ± 4.88

**Table 5:** Total amount of crude fat per 100 mL and total amount of crude fat being digested per 100 mL intake (calculated with the mean pc fat DR) in the different groups; in brackets the rank (1 highest, 4 lowest) is given for each column.

HCD	Crude Fat (g/100 mL)	Amount of Digested Crude Fat (g/100 mL)		
		CON	PL	PERT
A	9.30 (2)	8.98 (1)	4.41 (3)	9.20 (1)
C	9.35 (1)	8.93 (2)	5.07 (1)	9.09 (2)
D	5.80 (3)	5.55 (3)	4.87 (2)	5.64 (3)
F	4.90 (4)	4.73 (4)	2.59 (4)	4.73 (4)

**Table 6:** Labelled compounds of the commercial high-caloric liquid products designed for enteral nutrition to prevent malnutrition used in this study.

HCD	Fat Source	Emulsifier	Protein Source	Carbohydrate Source
A	rapeseed oil, sunflower oil	lecithin from soy	cow milk	glucose
B	rapeseed oil, sunflower oil, corn oil	lecithin from soy, E466	cow milk	hydrolysed corn starch, saccharose
C	rapeseed oil, sunflower oil	E471, lecithin from soy	cow milk	maltodextrin, sugar
D	rapeseed oil, corn oil, sunflower oil	E471	cow milk	glucose, saccharose

**Table 7:** Total amount of crude protein per 100 mL and total amount of crude fat being digested per 100 mL intake (calculated with the mean pc protein DR) in the different groups; in brackets, the rank (1 highest, 4 lowest) is given for each column.

HCD	Crude Protein (g/100 mL)	Amount of Digested Crude Protein (g/100 mL)		
		CON	PL	PERT
A	9.60 (2)	7.38 (2)	2.90 (1)	7.36 (2)
B	10.2 (1)	7.86 (1)	2.29 (2)	8.14 (1)
C	5.60 (4)	4.40 (4)	1.88 (4)	4.22 (4)
D	6.30 (3)	4.42 (3)	2.01 (3)	4.96 (3)

## Data Availability

The original contributions presented in this study are included in the article/supplementary material. Further inquiries can be directed to the corresponding author(s).

## Abbreviations

Con	Control
DR	Disappearance rate
HCD	High-calorific drink
pcDR	Praecaecal disappearance rate
PEI	Pancreatic exocrine insufficiency
PERT	Pancreatic enzyme replacement therapy
PL	Pancreatic duct-ligated
PL + PERT	Pancreatic duct-ligated individual receiving pancreatic enzyme replacement therapy
